# Vaccination of mice with *Trichinella spiralis* C-type lectin elicited the protective immunity and enhanced gut epithelial barrier function

**DOI:** 10.1371/journal.pntd.0012825

**Published:** 2025-01-22

**Authors:** Bo Ning Wang, Xin Zhuo Zhang, Jin Yi Wu, Zhao Yu Zhang, Pei Kun Cong, Wen Wen Zheng, Shao Rong Long, Ruo Dan Liu, Jing Cui, Zhong Quan Wang

**Affiliations:** Department of Parasitology, School of Basic Medical Sciences, Zhengzhou University, Zhengzhou, China; Uniformed Services University: Uniformed Services University of the Health Sciences, UNITED STATES OF AMERICA

## Abstract

**Background:**

C-type lectin (CTL) plays an important act in parasite adhesion, host’s cell invasion and immune escape. Our previous studies showed that recombinant *Trichinella spiralis* C-type lectin (rTsCTL) mediated larval invasion of enteral mucosal epithelium. The aim of this study was to investigate protective immunity produced by vaccination with rTsCTL and its effect on gut epithelial barrier function in a mouse model.

**Methodology/principal finding:**

The ELISA results showed that subcutaneous vaccination of mice with rTsCTL elicited a systemic humoral response (high levels of serum IgG, IgG1/IgG2a and IgA) and significant gut mucosal sIgA responses. The levels of Th1/Th2 cytokines (IFN-γ/IL-4) secreted from spleen, mesenteric lymph nodes and Peyer’s patches were distinctly increased at 6 weeks following vaccination (*P* < 0.05). At one week after challenge, the numbers of goblet cells and expression level of Muc2, Muc5ac and pro-inflammatory cytokines (TNF-α and IL-1β) in gut tissues of vaccinated mice were obviously decreased, while expression of anti-inflammatory cytokines (IL-4 and IL-10) was evidently increased, compared to the infected PBS group. It is interesting that expression levels of gut epithelial tight junctions (TJs; occludin, claudin-1 and E-cad) were prominently elevated and intestinal permeability was interestingly declined in vaccinated mice. The rTsCTL-vaccinated mice exhibited a 51.69 and 48.19% reduction of intestinal adult and muscle larva burdens, respectively. The female fecundity in rTsCTL vaccinated mice was reduced by 40.51%. These findings indicated that rTsCTL vaccination impeded larval invasion and improved gut epithelial integrity and barrier function, reduced worm burdens, and relieved gut and muscle inflammation.

**Conclusions:**

Vaccination of mice with rTsCTL elicited an obvious protective immunity against larval challenge, impeded larval invasion of gut mucosa, enhanced gut epithelial integrity and barrier function, reduced worm burdens; it also alleviated gut and muscle inflammation. TsCTL might be a novel candidate target molecule for anti-*Trichinella* vaccines.

## Author summary

C-type lectin (CTL) plays a vital role in parasite adhesion, invading host’s cells and immune escape. Previous studies showed that recombinant *Trichinella spiralis* C-type lectin (rTsCTL) facilitated larval invasion of intestinal epithelium. The purpose of this study was to investigate the protective immunity evoked by vaccination with rTsCTL in a mouse model. The results revealed that subcutaneous vaccination of mice with rTsCTL elicited a systemic humoral response (high levels of serum IgG, IgG1/IgG2a and IgA) and significant gut mucosal sIgA responses. The levels of Th1/Th2 cytokines (IFN-γ/IL-4) secreted from spleen, mesenteric lymph nodes and Peyer’s patches were distinctly increased at 6 weeks following vaccination (*P* < 0.05). The rTsCTL-vaccinated mice exhibited a 51.69 and 48.19% reduction of intestinal adult and muscle larva burdens, respectively. Moreover, the numbers of goblet cells and expression level of Muc2, Muc5ac and pro-inflammatory cytokines (TNF-α and IL-1β) in gut tissues of vaccinated mice were obviously decreased after challenge, while expression of anti-inflammatory cytokines (IL-4 and IL-10) was evidently increased. Additionally, expression levels of tight junctions (occludin, claudin-1 and E-cad) were prominently elevated and intestinal permeability was interestingly declined in vaccinated mice. The results showed that vaccination of mice with rTsCTL elicited an obvious protective immunity against *T. spiralis* challenge, impeded larval invasion of the gut, reduced intestinal adult burden, female fecundity and muscle larval burdens; it also alleviated gut inflammations, consequently improved intestinal epithelial integrity and enhanced intestinal mucosal barrier function. Therefore, TsCTL might be a novel candidate target molecule for anti-*Trichinella* vaccines.

## Introduction

Trichinellosis is a worldwide foodborne zoonotic parasitic disease caused by the nematode *Trichinella* spp. which can infect over 150 kinds of mammals, birds, reptiles and humans around the world. *Trichinella* infection is caused by consumption of raw or poorly cooked animal meat containing *T. spiralis* infectious muscle larvae (ML). The domestic pig pork is the principal source of human trichinellosis in China and other developing countries [1]. Only in 2022, 41 confirmed cases of human trichinellosis were reported in the 22 member states of the EU [2]. In China, eight trichinellosis outbreaks with 479 cases and 2 deaths were recorded from 2009 to 2020, and seven outbreaks (87.50%) were involved in the ingestion of raw or semi-cooked pork and pork products [3]. Trichinellosis is not only a severe public health problem, but also a main risk for meat food safety [4]. Therefore, it is essential to develop a preventive vaccine to interrupt *Trichinella* infection in food animals and to eliminate the ML from animal meat food [5,6].

After animal meat containing the encapsulated ML is ingested, the collagen capsules are digested by gastric fluids and the larvae are released. The larvae are activated into intestinal infectious larvae (IIL) by intestinal contents or bile [7]. The IIL invades intestinal epithelium cells (IECs) and undergoes 4 molts within approximately 31 hours after infection to develop into adult worms (AW). After mating, the pregnant female adults produce the newborn larvae (NBL). And then, the NBL migrate along with the venous and lymphatic systems, when they invade skeletal muscle cells the larvae are encapsulated to complete the lifecycle. The IIL invasion of IECs is the first crucial stage in the process of *T. spiralis* infection in the intestines. The gut epithelium serves as the primary physical defense barrier against the intrusion of *T. spiralis* and is the dominant site of interaction between the parasite and the host [8,9]. The mucosal immune response plays a vital role in the process of immunity against *Trichinella* IIL invasion and development, and worm expulsion from intestinal tract. The ideal anti-*Trichinella* vaccines should be capable of impeding IIL invasion of gut mucosa, interrupting the development of the IIL to the AW, discharging IIL and AW from the gut, suppressing female fecundity, destroying the residual and escaped larvae in muscle tissues [10–13].

C-type lectin (CTL) is one of the largest families of lectins and is ubiquitous in bacteria, vertebrate and invertebrate animals. CTL binds carbohydrates dependent on Ca^2+^ [14]. It has been found that the CTL has one or more C-type lectin domains (CTLD). The CTLD is also called carbohydrate-recognition domains (CRD). CTL binds with various ligands, such as carbohydrates, lipids, proteins, and inorganic matter [15]. Previous research showed that parasite lectins are important for binding glycans on the surface of host cells, helping parasite adhere to host cells and mediating parasite recognition and activation of host immune responses [16]. *Toxoplasma gondii* invasion of host cells was facilitated by *T. gondii* lectin CD209 through their interaction, which was hindered by mimicking oligosaccharides and anti-CD209 antibody [17]. *Cryptosporidium parvum* CTL facilitates the attachment and infection of the parasites by binding to heparan sulfate proteoglycans (HSPG) on the IECs in a Ca^2+^-dependent manner [18].

In previous study, a new C-type lectin domain-containing protein from *T. spiralis* (TsCTL; GenBank: KRY42391.1) was identified and expressed in our department [19], natural TsCTL was highly expressed at the IIL stage. rTsCTL binding IECs specifically mediated the IIL intrusion of IECs, while anti-rTsCTL antibodies and mannose inhibited the IIL invasion [20]. Moreover, a further study showed that rTsCTL binding to syndecan-1 in Caco-2 cells activated the STAT3 pathway, reduced expression of intestinal epithelial tight junctions (TJs), impaired the integrity of intestinal epithelium barrier, and mediated the *T. spiralis* larval penetration of intestinal mucosa [21]. These results suggested that TsCTL might be a promising molecule target of preventive vaccines against *T. spiralis* invasion and infection.

The purpose of this study was to investigate gut local mucosal and systemic immune responses and protection produced by vaccination with rTsCTL in a model of BALB/c mice.

## Materials and methods

### Ethics statement

This study was performed in the light of National Guidelines for Experimental Animal Welfare (Minister of Science and Technology, People’s Republic of China, 2006). All animal experiments in this study were approved by the institutional Life Science Ethics Committee of Zhengzhou University (No. ZZUIRB GZR 2022-1317).

### *Trichinella* species and experimental animal

*Trichinella spiralis* (ISS534) was collected from an infected pig in Henan province of China and preserved by serial passage in BALB/c mice in our department. The female mice with 4–6 weeks old were purchased from the Experimental Animal Center of Zhengzhou University.

### Preparation of rTsCTL

Recombinant expression plasmid pQE-80L/TsCTL was constructed in our laboratory and used as an amplification template [19]. The TsCTL gene sequence consisted of 627 bp encoding 208 amino acids (aa), with a molecular weight (MW) of 24 kDa. The full-length TsCTL cDNA sequence was amplified using PCR by specific primers with *BamH I* and *Pst I* restriction enzyme sites **(bold and italicized**). The specific primers were 5ʹ-C***GGATCC***AACCGTTTTCCGTGC CGTATCAAAT3ʹ, 5ʹ-ACGC***GTCGAC***TCACTCCAACGAATGACAAATTC-3ʹ. The PCR products were cloned into the pQE-80L with N-terminus His-tag, and the recombinant plasmid pQE-80L/TsCTL was transferred into *Escherichia coli* BL21 (DE3) [22]. After being induced with 0.4 mM isopropyl β-d-1-thiogalactopyranoside (IPTG) at 25 °C for 8 h, rTsCTL was expressed, and purified using a Ni–NTA His-tag affinity kit (Novagen, USA) [23] and identified by SDS-PAGE and Western blot as previously described [21,24].

### Immunization of mice with rTsCTL and sample collection

Total of 120 mice were randomly divided into 3 groups (40 mice per group). Each group of mice was subcutaneously injected by using 20 µg rTsCTL [25]. The rTsCTL proteins were pre-emulsified with ISA 201 adjuvant (SEPPIC, France). The mice were boosted two times with the same amount of rTsCTL emulsified with ISA 201 adjuvant at a 2-week-interval. Control groups were administered with only ISA 201 adjuvant or PBS. Two weeks after the final vaccination, all mice were orally challenged with 300 *T. spiralis* muscle larvae (ML).

**Fig 1:**
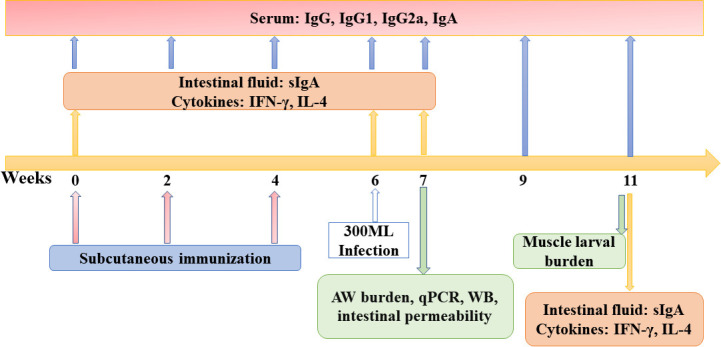
The designed vaccination scheme and detection protocol. Subcutaneous immunization of mice was administered three times (weeks 0, 2 and 4). The vaccinated mice were orally challenged with 300 *T*. *spiralis* ML two weeks following the final vaccination. Anti-rTsCTL antibodies (IgG, IgG1, IgG2a and IgA) were measured by ELISA using rTsCTL at week 0, 2, 4, 6 after first immunization, respectively, and at week 1, 3, 5 following challenge infections. Five mice of each group were sacrificed before immunization, 6 weeks after immunization, and 7 and 35 dpi; the levels of sIgA and cytokines (IFN-γ and IL-4) were assessed. At 7 and 35 dpi, ten mice of each group were sacrificed and intestinal adult worm, female fecundity and muscle larval burden (larvae per gram, LPG) were respectively ascertained to evaluate the immune protection elicited by vaccination with rTsCTL. Histopathological examination of intestines and muscles from infected mice was performed at 7 and 35 dpi.

One hundred microliters of tail blood were obtained from ten mice of each group at weeks 0, 2, 4, 6, 7, 9 and 11 after the first vaccination, serum samples were isolated and stored at −80 °C until use [26]. Five mice of each group were sacrificed at weeks 0, 6, 7 and 11 weeks after vaccination, and the intestine, spleens, mesenteric lymph nodes (MLNs) and Peyer’s patches (PPs) were collected to ascertain intestinal sIgA and cytokine responses. To assess immune protective efficacy of rTsCTL vaccination, additional 10 mice of each group were respectively euthanized at weeks 7 and 11 after vaccination, e.g., 7 days post infection (dpi) and 35 dpi. The adult worm (AW) burden, female reproductive capacity (the in vitro production of newborn larvae (NBL) deposited by each female for 72 h), and ML burden were ascertained as previously described [6]. Moreover, an additional blank control group (5 unvaccinated and uninfected mice) was set up in assay of intestinal permeability, goblet cell numbers and gene expression of the gut epithelial tight junctions and mucins. The scheme of vaccination protocol was shown in Fig 1.

### ELISA determination of serum anti-rTsCTL antibodies

Serum specific anti-rTsCTL IgG (IgG1/IgG2a) and IgA were measured in all vaccinated mice by indirect ELISA using rTsCTL as coating antigens [5]. Briefly, ELISA plate was coated with 2 μg/ml rTsCTL at 4 °C overnight. After being washed with PBS containing 0.5% Tween (PBST), the plate was blocked with 5% skimmed milk in PBST for 2 h at 37 °C. Following washes again, the plate was probed with 1:100 dilutions of murine immune sera at 37 °C for 1 h, and then incubated with HRP-conjugated anti-mouse IgG, IgG1/IgG2a, IgA (1:10000; Sigma, USA) at 37 °C for 1 h, then colored using the substrate o-phenylenediamine dihydrochloride (OPD; Sigma) plus 0.15% H_2_O_2_, and the reaction was stopped by using 2 M H_2_SO_4_. The absorbance (OD value) at 492 nm was assayed by a microplate reader (Tecan, Schweiz, Switzerland) [27].

### Assessment of intestinal total sIgA and rTsCTL-specific sIgA

To ascertain total and rTsCTL-specific sIgA in gut fluid, enteric washing was collected as described previously [28,29]. Briefly, a 20 cm long small intestine was excised, and the content was washed three times with 1 ml of cold PBS containing 1% protease inhibitor (Sangon Biotech., Shanghai, China). The eluting fluid was collected and centrifuged at 12,000 *g* for 5 min at 4 °C, and then supernatant was collected and stored. Enteric fluid was diluted at 1:10 when used. Total gut sIgA was measured by a sandwich ELISA, and rTsCTL-specific sIgA was detected by an indirect ELISA with 2 μg/ml of rTsCTL. Coloration with OPD and absorbance at 492 nm were determined as depicted before [30].

### ELISA determination of cytokine responses

To assess the cellular immune response to the rTsCTL vaccination, five mice of each group were euthanized at week 0 and 6 following vaccination, and at 1 and 5 weeks after challenge. The spleen, MLNs and PPs were recovered from all vaccinated mice, homogenized in complete DMEM medium (Gibco, Auckland, New Zealand). The cells were obtained after centrifugation at 1000 *g* for 5 min, and isolated as reported [5,31]. The cell density was adjusted to 2 × 10^6^ cells/ml in DMEM medium containing 5% fetal bovine serum (FBS), penicillin (100 U/ml) and streptomycin (100 μg/ml), stimulated with 10 μg/ml rTsCTL at 37 °C and 5% CO_2_ for 72 h [32], then supernatant was collected and two cytokines (IFN-γ and IL-4) were measured by a sandwich ELISA (BD Biosciences Pharmingen, USA). The levels of cytokine were shown as pictograms per milliliter (pg/ml).

### Challenge infection and evaluation of immune protection

In order to evaluate immune protection produced by rTsCTL vaccination, all vaccinated mice were challenged orally with 300 *T. spiralis* ML two weeks after the last vaccination. The AWs were collected and numbered from the intestines of ten vaccinated mice from each group at 7 dpi. The remaining ten mice from each group were euthanized at 35 dpi, mouse carcass was weighted, artificially digested and the ML were collected and numbered as reported before [12,33]. The immune protective effect induced by rTsCTL vaccination was assessed as the worm burden reduction of enteral AWs and muscle larvae per gram (LPG) of skeletal muscle tissues from immunized mice compared to the PBS group [11]. Additionally, the female fecundity from various groups of vaccinated mice was also ascertained as previously reported [34].

### Antibody-dependent cell-mediated cytotoxicity (ADCC) assay

Specific antibody mediated cytotoxicity on the NBL was performed as previously reported [13]. Briefly, The female adult worms at 6 dpi were cultivated in DMEM with 10% fetal bovine serum (FBS; Gibco) at 37 °C in 5% CO_2_ for 24 h, and the NBL were recovered, 100 NBL were cultured with 2 × 10^5^ murine peritoneal exudate cells (PECs) in a 96-well plate with DMEM medium supplemented with anti-rTsCTL immune serum (1:50–1:800 dilutions) at 37 °C for 72 h, *T. spiralis-*infected mouse serum was used as positive control, mouse serum from the ISA 201 and PBS control groups as negative serum controls. After being cultured for 72 h, the NBL viability was assessed according to their morphology and activity. The living NBL was active and mobile, while the dead NBL was inactive and straight. Cytotoxicity was defined as the percentage of dead NBL to the total larvae observed in each test.

### Assay of intestinal permeability in infected mice

In order to evaluate whether rTsCTL immunization affects intestinal permeability in infected mice, intestinal permeability assay was performed in immunized and infected mice [35,36]. In brief, at 7 dpi, all mice were fasted and were deprived from water overnight, and then 100 μl 4 kDa FITC-dextran (FD 4) was administrated to each mouse at a concentration of 50 mg/ml by intragastric administration. And then the mice recovered drinking water. Four hours later, mouse blood was collected and blood plasma was isolated in the dark. The plasma was diluted in a 1:100 ratio with PBS, and the absorbance at 485 nm excitation wavelength and 520 nm emission wavelengths were measured by a microplate reader (Tecan, Switzerland) [37,38].

### qPCR and Western blot assay of TJs, mucins and inflammatory cytokines of gut mucosa from infected mice

To detect mRNA expression levels of TJs (occludin, claudin-1, claudin-2 and E-cad), Muc2, Muc5, pro-inflammatory cytokines (TNF-α and IL-1β) and anti-inflammatory cytokines (IL-4 and IL-10), mouse intestinal tissues were collected at 7 dpi, total RNAs were extracted with TRIzol reagent (Takara), and were reverse-transcribed to cDNA using a cDNA synthesis kit [39]. qPCR amplification was performed using the SYBR Green PCR master mix in the ABI Prism 7500 Fast Sequence Detection System (Applied Biosystems, South San Francisco, USA) [40]. β-actin was used to normalize mRNA levels, and there were no differences in β-actin expression among different groups. A PBS negative control was set on each experiment. The fold changes in the nine genes were calculated using the comparative Ct (2^−ΔΔCt^) method [41]. Each experiment was carried out in triplicate. Primers of nine genes used for qPCR in this study are listed in Table 1 [8,42].

**Table 1:** Primer sequences of murine TJs, mucin and cytokines in qPCR assays.

Genes	Sequences (5ʹ to 3ʹ)	GenBank no.
Occludin	F: TGGCAAGCGATCATACCCAGAG	NM_001360536.1
	R: CTGCCTGAAGTCATCCACACTC	
Claudin-1	F: GGACTGTGGATGTCCTGCGTTT	NM_016674.4
	R: GCCAATTACCATCAAGGCTCGG	
Claudin-2	F: AGGACTTCCTGCTGACATCCAG	NM_001410421.1
	R: AATCCTGGCAGAACACGGTGCA	
E-cad	F: GGTCATCAGTGTGCTCACCTCT	NM_009864.3
	R: GCTGTTGTGCTCAAGCCTTCAC	
Muc2	F: TGTGGCCTGTGTGGGAACTTT	NM_023566.4
	R: CATAGAGGGCCTGTCCTCAGG	
Muc5ac	F: CTGTGACATTATCCCATAAGCCC	NM_010844.3
	R: AAGGGGTATAGCTGGCCTGA	
TNF-α	F: CCCTCACACTCAGATCATCTTCT	NM_013693.3
	R: GCTACGACGTGGGCTACAG	
IL-1β	F: AGCTCTCCACCTCAATGGAC	NM_008361.4
	R: ATCATTGCGTGGGATCTTGA	
IL-4	F: TTGTCATCCTGCTCTTCTTTCT	NM_021283.2
	R: CTGTGGTGTTCTTCGTTGCT	
IL-10	F: CCCTTTGCTATGGTGTCCTT	NM_010548.2
	R: TGGTTTCTCTTCCCAAGACC	
β-actin	F: CTACCTCATGAAGATCCTGACC	NM_007393.5
	R: CACAGCTTCTCTTTGATGTCAC	

To ascertain the expression levels of TJs (occludin, claudin-1, claudin-2 and E-cad), mouse intestinal tissues at 7 dpi were lysed in RIPA buffer, and were grinded in an ice bath for 30 min and centrifuged at 12,000 *g* for 15 min to remove any cell fragments. The tissue proteins were separated by 10% SDS–PAGE and transferred onto a polyvinylidene difluoride (PVDF) membrane (Millipore, USA) in the wet transfer cell (Bio-Rad, USA) [43]. The membrane was blocked with 5% skim milk in TBST at 37 °C for 2 h and incised into strips. Subsequently, the strips were probed with antibodies against occludin (1.5 μg/ml), claudin-1 (1.5 μg/ml), claudin-2 (1.5 μg/ml) (ThermoFisher, USA), E-cad (1: 1000, Abmart, China) and anti-β-actin antibody (1:1000, Servicebio, Wuhan, China) overnight at 4 °C [44]. After washes with TBST, the strips were incubated at 37 °C for 1 h with HRP-anti-mouse IgG conjugate or HRP-conjugated anti-rabbit IgG (1:10,000; Southern Biotech). Finally, the strips was colored with an enhanced chemiluminescence kit (Epizyme, Shanghai, China) and the relative intensities of each band were analyzed using the Image J software (National Institutes of Health, USA) [45,46].

### Histopathological examination of small intestine and muscle

At 7 and 35 dpi, small intestine and masseter muscles were respectively collected from infected mice and blank control mice, fixed in 4% polyoxymethylene for 24 h, embedded in paraffin wax and cut into 3-μm-thick tissue cross-sections, deparaffinized and stained using hematoxylin and eosin (HE) stain and periodic acid Schiff reagent (PAS; Baso, Zhuhai, China) [25,30]. Gut mucosa of different groups of mice were examined under light microscopy, and enteral villus width and the numbers of enteral epithelial goblet cells per field (400×) were examined and numbered. The encapsulated larvae per field (100×) and inflammatory cells (eosinophils, neutrophils and lymphocytes) per field (400×) on muscle sections were numbered as previously described [13,47].

### Statistical analysis

The data in this study were analyzed by GraphPad Prism V.9.5 (GraphPad Software Inc., San Diego, CA, USA) and shown as the mean ± standard deviation (SD). Differences among diverse groups were analyzed by Student’s t-test or one way ANOVA after being tested by Shapiro-Wilk’s test and Levene’s test to check the datum normality and homogeneity. *P* < 0.05 was regarded as statistical significance.

## Results

### Serum anti-rTsCTL antibodies in immunized mice

**Fig 2:**
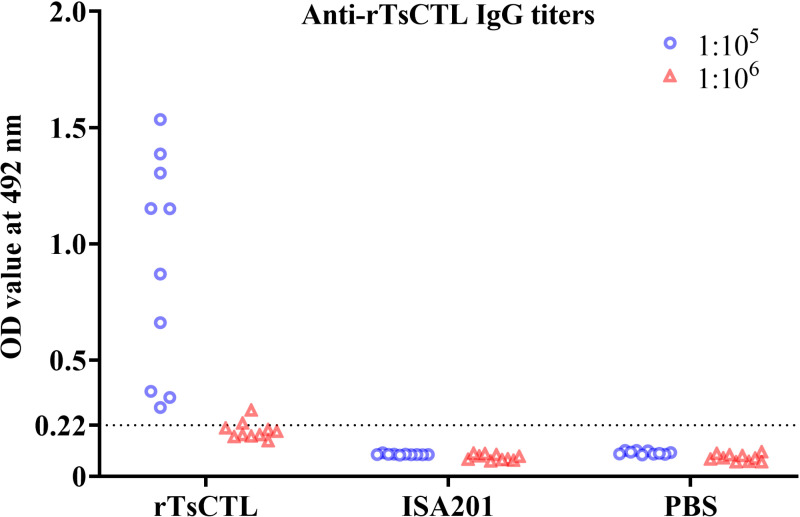
Serum anti-rTsCTL IgG titers measured by ELISA with rTsCTL. Anti-rTsCTL IgG levels were assayed in sera of vaccinated mice 2 weeks after the last vaccination. All serum samples were assayed in duplicate. The data are presented as the OD values of anti-rTsCTL IgG levels from 10 vaccinated mice. Forty five serum samples (1:100 dilutions) from normal mice were also measured as negative serum controls. The cut-off values of ELISA were calculated based on the 2.1-fold of the mean OD value of the negative control serum from normal mice. Serum OD values that were equal to or greater than the cut-off values were regarded as positive. The cut-off values (0.22) are shown with a dotted line.

Anti-rTsCTL IgG titers in murine sera were measured by ELISA at two weeks after the final vaccination. The results showed that anti-rTsCTL IgG levels in vaccinated mice were significantly increased compared to the pre-vaccination levels (*P* < 0.05). After the final vaccination, the specific IgG titer in vaccinated mice reached 1:10^5^ (Fig 2), indicating that rTsCTL had a good immunogenicity. However, anti-rTsCTL IgG responses were not detected in mice vaccinated with only ISA201 and PBS.

**Fig 3:**
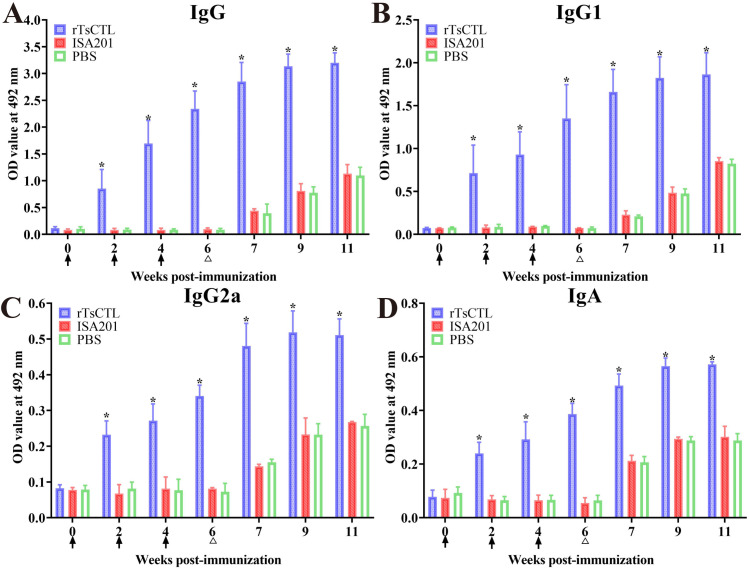
Serum anti-rTsCTL antibodies of immunized mice determined by ELISA using rTsCTL. **A:** Total anti-rTsCTL IgG response in mice vaccinated with rTsCTL at various times following vaccination. Specific IgG1 (**B**) and IgG2a (**C**) subclass responses were also ascertained at various times after vaccination. **D:** Specific IgA level in vaccinated mice. All serum samples were assayed in duplicate. The OD values from each group of mice are presented as mean ± standard deviation (SD) of antibody levels (n = 10). The vaccination times are shown with arrows (↑) and the challenge time is indicated by triangles (∆). *P < 0.05 compared to the PBS group.

The ELISA results showed that at 2, 4 and 6 weeks after first immunization, serum anti-rTsCTL IgG level of the rTsCTL group was significantly higher than the ISA201 and PBS groups (*F*_2W_ = 45.49, *F*_4W_ = 138.1, *F*_6W_ = 455.6, *P* < 0.0001), and remained higher level at 1–5 weeks after larval challenge (Fig 3A). Moreover, the IgG1 and IgG2a levels of rTsCTL group were also statistically higher than the ISA201 and PBS groups at 6 weeks after first immunization and 5 weeks after challenge (*F*_IgG1_ = 106.0, *F*_IgG2a_ = 471.0, *P* < 0.0001) (Fig 3B and 3C). Furthermore, the IgG1 level of rTsCTL group at 2, 4 and 6 weeks after first immunization was obviously higher than IgG2a level (*t*_2w_ = 4.633, *P* = 0.0012; *t*_4w_ = 7.265, *P* < 0.0001; *t*_6w_ = 7.815, *P* < 0.0001), indicating that rTsCTL immunization triggered a mixed Th1/Th2 immune response with Th2 predominance. Additionally, anti-rTsCTL IgA was also measured, the results showed that IgA levels at 2, 4 and 6 weeks following the first vaccination were visibly elevated in rTsCTL group compared to the ISA201 and PBS control groups (*F*_2W_ = 141.9, *F*_4W_ = 102.4, *F*_6W_ = 448.6, *P* < 0.0001) (Fig 3D). But the mice vaccinated with ISA201 or PBS alone did not exhibit any anti-rTsCTL IgG and IgA responses at 2, 4 and 6 weeks after vaccination; but after larval challenge, the two control groups also showed increasing anti-rTsCTL IgG and IgA level in comparison to pre-challenge levels. The results demonstrated that specific IgG (IgG1/ IgG2a) and IgA levels in immunized group were gradually elevated after vaccination, and further increased after challenge infection, and rTsCTL vaccination triggered obvious humoral immune responses.

### Intestinal mucosal sIgA response

**Fig 4:**
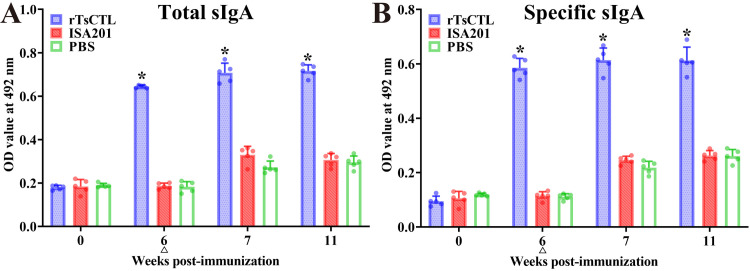
Total enteral sIgA (A) and rTsCTL specific sIgA (B) in enteric washing fluid of vaccinated mice. The data are shown as the mean OD values ± SD of five mice per group. All enteric fluid samples were assayed in duplicate. No evidently detectable sIgA response was observed in mice vaccinated with ISA201 or PBS control group. The challenge time is indicated by a triangle (∆). *P < 0.05 compared to the PBS group.

The results of sIgA assay revealed that total sIgA level in gut fluid of mice immunized with rTsCTL were evidently higher than the PBS group at 6 weeks after the first immunization (*F* = 1294, *P* < 0.0001) (Fig 4A). Moreover, rTsCTL-specific sIgA levels in rTsCTL group were also distinctly higher than the PBS group (*F* = 696.6, *P* < 0.0001) (Fig 4B). The higher levels of total and specific sIgA in rTsCTL group sustained to the 5 weeks after the challenge (*F*_total_ = 335.3, *F*_specific_ = 182.7, *P* < 0.0001). No enteral mucosal specific sIgA responses were observed in mice vaccinated with the only ISA201 or PBS alone group. The finding suggested that rTsCTL vaccination elicited an evident enteric mucosal sIgA response.

### Cytokine expression levels of immunized mice

**Fig 5:**
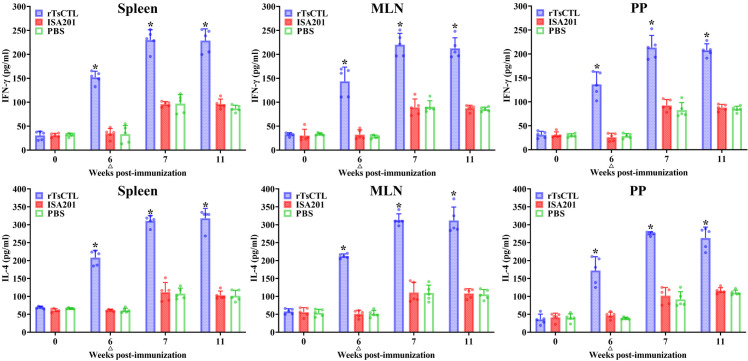
Cytokines secreted by spleen, mesenteric lymph nodes (MLN) and Peyer’s patches (PP) from immunized mice with the rTsCTL at different times after vaccination. Concentrations of two cytokines (IFN-γ and IL-4) were measured in supernatant after the spleen, MLN and PP cells were stimulated with 10 μg of rTsCTL for 72 h at 37 °C and 5% CO_2_. The data are shown as the mean pictograms per milliliter (pg/ml) ± SD of five mice per group. The challenge time is indicated by a triangle (∆). All samples were assayed in duplicate. *P < 0.05 compared to the PBS group.

To investigate Th1/Th2 response, splenocytes, MLN and PP from immunized mice were stimulated with the rTsCTL, the cytokine profile from culture supernatants was assayed by ELISA. The results showed that the levels of Th1 cytokine (IFN-γ) and Th2 cytokine (IL-4) in the rTsCTL group of mice had no significant difference before immunization (*P* > 0.05). But the level of IFN-γ and IL-4 secreted by spleen cells in rTsCTL immunization group were remarkably increased at 6 weeks after immunization, compared to the PBS group (*F*_IFN-γ_ = 117.1, *F*_IL-4_ = 235.0, *P* < 0.0001) (Fig 5). Moreover, the levels of IFN-γ and IL-4 in rTsCTL group were further elevated at five weeks after challenge (11 weeks following vaccination) (*F*_IFN-γ_ = 124.5, *F*_IL-4_ = 202.4, *P* < 0.0001). The levels of IFN-γ and IL-4 secreted by MLN (6w: *F*_IFN-γ_ = 64.22, *F*_IL-4_ = 564.8, *P* < 0.0001; 11w: *F*_IFN-γ_ = 148.1, *F*_IL-4_ = 118.1, *P* < 0.0001) and PP cells (6w: *F*_IFN-γ_ = 77.67, *F*_IL-4_ = 52.33, *P* < 0.0001; 11w: *F*_IFN-γ_ = 283.7, *F*_IL-4_ = 107.9, *P* < 0.0001) were also evidently higher than the PBS group at 6 weeks following immunization and 5 weeks after challenge. The results suggested that vaccination with rTsCTL triggered the concomitant Th1/Th2 responses, and indicated that subcutaneous immunization with rTsCTL evoked both systemic (spleen) and intestinal mucosal local (MLN and PP) cellular immune responses.

### Immune protection of immunization with rTsCTL

**Fig 6:**
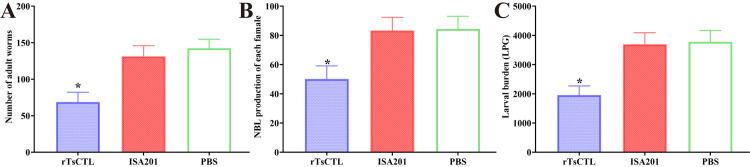
Immune protection induced by immunization with rTsCTL following challenge with 200 *T. spiralis* larvae in a murine model. **A:** Intestinal adult worm burdens. **B:** The *in vitro* production of NBL deposited by each female in 72 h (n = 30). **C:** Muscle larval burden (larvae per gram, LPG). The worm burdens are presented as mean ± SD from ten mice per group. *P < 0.05 compared to the PBS group.

Immune protection against *T. spiralis* larval challenge was investigated in all vaccinated mice. The results showed that compared to the PBS group, rTsCTL vaccination group exhibited 51.69% intestinal AW reduction at 7 dpi following larval challenge (*F* = 88.06, *P* < 0.0001) (Fig 6A). Three females/each mouse were randomly collected from three mice of each group and cultured in DMEM at 37 °C for 72 h to calculate the NBL production per female worm. The results showed that female fecundity of the rTsCTL group was evidently lower than that of the PBS group. The NBL number in the rTsCTL group was reduced by 40.51% (*F* = 145.6, *P <* 0.0001) (Fig 6B). Moreover, vaccination of mice with rTsCTL showed a 48.19% reduction of the ML burden at 35 dpi (*F* = 78.70, *P* < 0.0001) (Fig 6C). However, vaccination of mice with only ISA 201 adjuvant did not show any evident reduction of intestinal adult worm and muscle larva burdens compared to the PBS group (*P* > 0.05). The results demonstrated that vaccination of mice with rTsCTL elicited an obvious immune protection against *T. spiralis* challenge infection, which reduced intestinal worm burden, impeded intestinal worm development and reduced female reproductive capacity, therefore, reduced the muscle larva burden and alleviated *T*. *spiralis* infection in immunized mice.

### ADCC killing and destroying on the NBL

**Fig 7:**
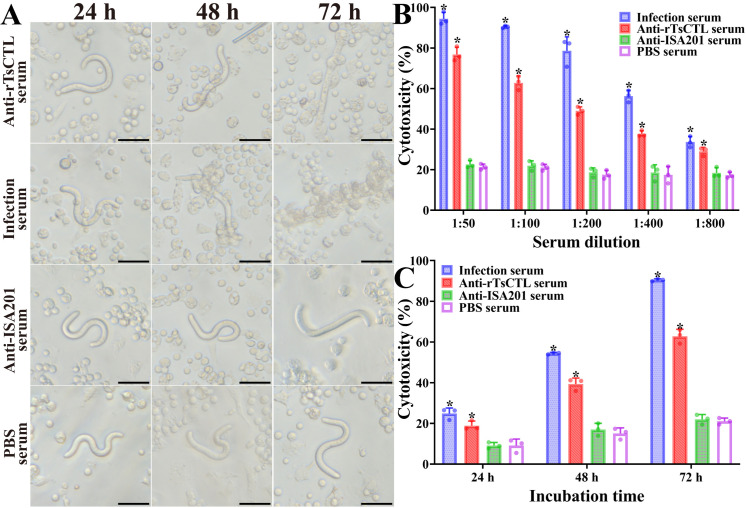
Anti-rTsCTL antibody mediated the ADCC killing on the NBL. In the test, the NBL were incubated with various sera and 2 × 10^5^ mouse peritoneal exudate cells (PECs). Various immune sera mediated killing effects on NBL at different incubation times. **A:** Anti-rTsCTL serum (1:100 dilutions) mediated the PECs adhesion to the NBL and destroyed the NBL. Infection serum was used as positive control. Sera from the ISA201 and PBS groups used as negative control. Scale bars = 50 μm. **B:** Cytotoxicity was dose-dependent of specific anti-rTsCTL antibodies. **C:** The cytotoxicity had an elevating trend with prolongation of culture time when 1:100 dilutions of anti-rTsCTL serum were used. Each test was performed in triplicate. *P < 0.05 compared to the PBS group.

The ADCC results revealed that after culture at 37 °C for 72 h, anti-rTsCTL serum (1:100 dilutions) mediated the PECs adhesion to the NBL and damage of the NBL (Fig 7A). When 1:100 dilutions of anti-rTsCTL serum were supplemented into the medium and were co-cultured with the NBL as well as PECs for 72 h, the ADCC resulted in a 62.85% cytotoxicity (NBL death), which were evidently higher than the sera from the ISA201 and PBS groups (*F* = 685.8, *P* < 0.0001) (Fig 7B). The cytotoxicity was dose-dependently related with specific anti-rTsCTL antibodies (*r*_rTsCTL_ = 0.9064, *P* < 0.0001). Moreover, when 1:100 dilutions of anti-rTsCTL serum were used, the cytotoxicity showed an elevating trend with the prolongation of culture time (*F*_24h_ = 28.62, *P* = 0.001; *F*_48h_ = 166.6, *F*_72h_ = 685.8, *P* < 0.0001) (Fig 7C).

### rTsCTL immunization reduced the increased intestinal permeability caused by *T. spiralis* infection

**Fig 8:**
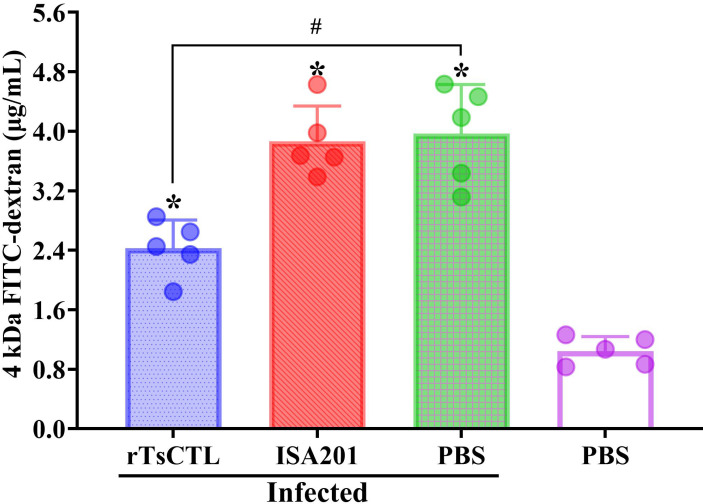
rTsCTL immunization reduced intestinal permeability in infected mice. Intestinal FD-4 flow was decreased in rTsCTL immunized mice after *T. spiralis* challenge infection (n = 5). * *P* < 0.05 compared with the uninfected PBS group; ^#^*P* < 0.05 contrast to the infected PBS group.

FD-4 was administered to each mouse at 7 dpi, and plasma was collected for intestinal permeability assay at 4 h after administration. The results showed that FD-4 flux in the rTsCTL group was reduced by 38.82% compared with the infected PBS group (*F* = 13.77, *P* = 0.0008) (Fig 8). The results showed that rTsCTL immunization reduced and abrogated the increase of intestinal permeability caused by *T. spiralis* infection and improved the integrity of intestinal epithelium, which might be related with rTsCTL immunization impeding larval invasion and reducing gut inflammation.

### rTsCTL immunization increased TJs expression in infected mice

**Fig 9:**
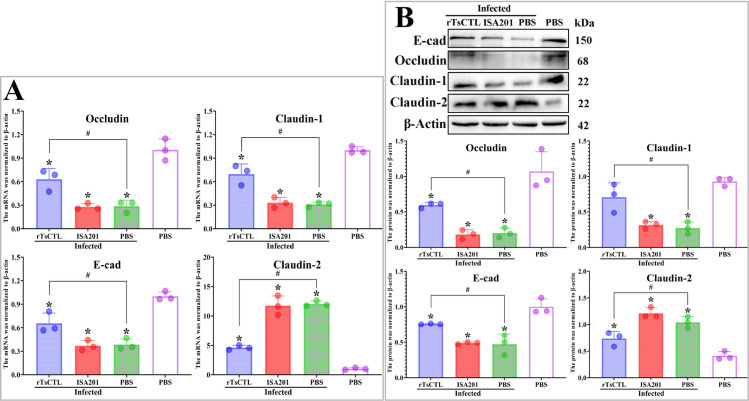
rTsCTL immunization increased the TJs expression levels in infected mice. **A:** qPCR analysis of mRNA expression levels of TJs (occludin, claudin-1, E-cad and claudin-2) in infected mice**. B:** Western blotting of protein expression levels of TJs (occludin, claudin-1, E-cad and claudin-2) in infected mice. β-actin was used as an internal reference. Each test had three replicates. * *P* < 0.05 compared with the uninfected PBS groups; ^#^*P* < 0.05 contrast to the infected PBS group.

The qPCR results showed that the transcription levels of occludin, claudin-1 and E-cad in the rTsCTL group were significantly higher than ISA201 or infected PBS groups at 7 dpi (*F*_occludin_ = 13.67, *P* < 0.01; *F*_claudin-1_ = 19.34, *P* < 0.01; *F*_E-cad_ = 8.610, *P* < 0.05) (Fig 9A); However, the claudin-2 transcription levels in the rTsCTL group were evidently lower than the ISA201 or infected PBS groups at 7 dpi (*F*_claudin-2_ = 51.55, *P* < 0.001).

Western blot results showed that the expression levels of occludin, claudin-1 and E-cad in the rTsCTL group at 7 dpi were obviously higher than the ISA201 or infected PBS groups (*F*_occludin_ = 46.85, *P* < 0.001; *F*_claudin-1_ = 10.15, *P* < 0.05; *F*_E-cad_ = 11.20, *P* < 0.01) (Fig 9B); Nevertheless, the claudin-2 expression levels in the rTsCTL group at 7 dpi were distinctly less than the ISA201 or infected PBS groups (*F*_claudin-2_ = 13.44, *P* < 0.01). These findings showed that after rTsCTL immunization, the decreased TJs expression levels resulted from *T. spiralis* infection was abrogated and regained, and indicated that rTsCTL immunization improved and strengthened intestinal epithelial integrity.

### Intestinal histopathological change in infected mice

**Fig 10:**
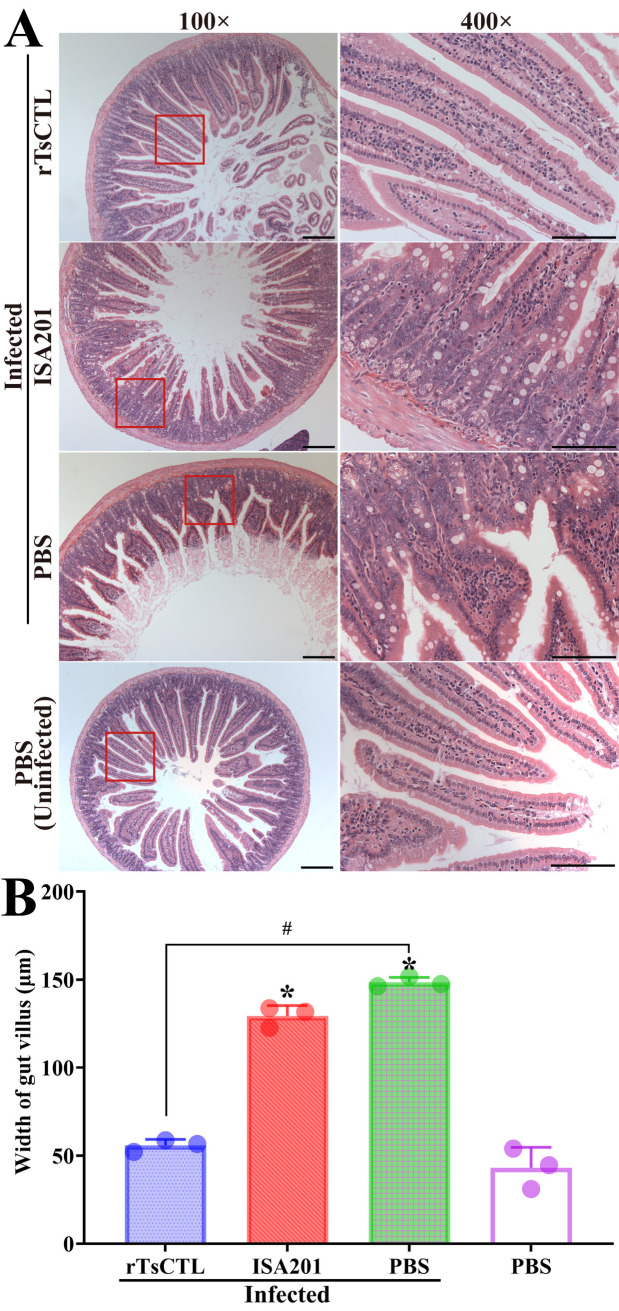
HE staining of intestinal histopathological changes in infected mice at 7 dpi. **A:** Enteral sections were stained by HE staining and observed on microscopy. Intestinal pathological changes from mice immunized with rTsCTL were significantly alleviated. Serious intestinal mucosal inflammation, shortened and edematous intestinal villi were observed in intestinal section of the infected ISA201 and PBS groups. **B:** Intestinal villus width of various groups of mice. Scale bars = 200 μm. Each test had three replicates. *P < 0.05 compared to the uninfected PBS group; ^#^*P* < 0.05 contrast to the infected PBS group.

**Fig 11:**
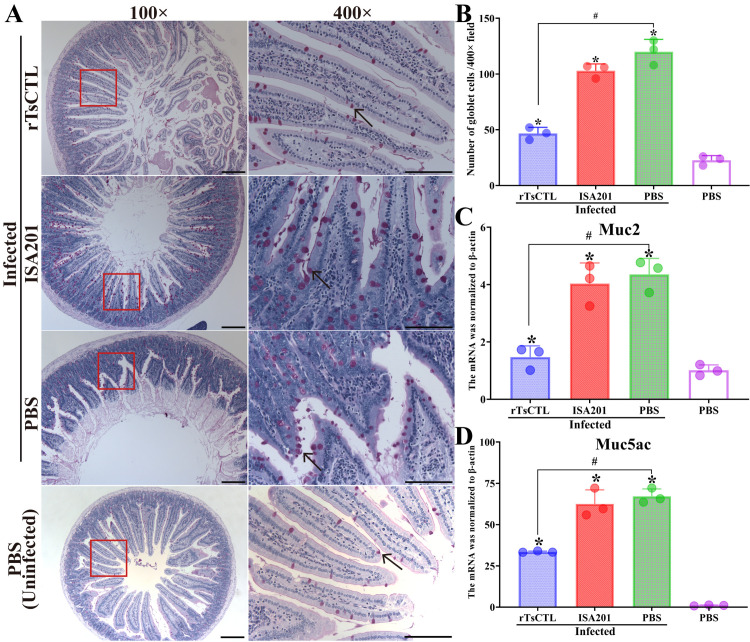
PAS staining of intestinal sections and mucin mRNA expression in infected mice at 7 dpi. **A:** Enteral sections were stained by PAS and examined on microscopy. The number of goblet cells of mice immunized with rTsCTL was obviously reduced compared with the infected ISA 201 and PBS control groups. Goblet cells were indicated by black arrows. **B:** Number of intestinal goblet cells/400× field. **C:** Relative expression level of Muc2 mRNA. **D:** Relative expression level of Muc5ac mRNA. Scale bars = 200 μm. Each test had three replicates. *P < 0.05 compared to the uninfected PBS group; ^#^*P* < 0.05 contrast to the infected PBS group.

The results of HE and PAS staining revealed that at 7 dpi, mild intestinal inflammation and relative normal intestinal villi were observed in enteral cross-sections of mice immunized with rTsCTL, intestinal villus width of the rTsCTL group was significantly lower than that of the ISA201 and infected PBS groups (*F* = 394.5, *P* < 0.0001) (Fig 10A and 10B), while enteral sections from the ISA201 and infected PBS groups showed an obvious destruction of villus structure, villous edema and inflammatory cell infiltration in the villus, and more as well as larger goblet cells (Fig 11A). The goblet cell numbers of the rTsCTL group were prominently less than the ISA201 and infected PBS groups (*F* = 69.30, *P* < 0.0001) (Fig 11B). Moreover, the qPCR results showed that Muc2 and Muc5ac transcription levels of the rTsCTL group were notably lower than those of the ISA201 and infected PBS groups (*F*_Muc2_ = 23.06, *P* < 0.01; *F*_Muc5ac_ = 32.29, *P* < 0.001) (Fig 11C and 11D). The results demonstrated that rTsCTL immunization significantly hampered larval invasion of gut mucosa, alleviated intestinal inflammation, and reduced mucin expression levels in gut mucosa.

### Inflammatory cytokine mRNA expression in infected mice

**Fig 12:**
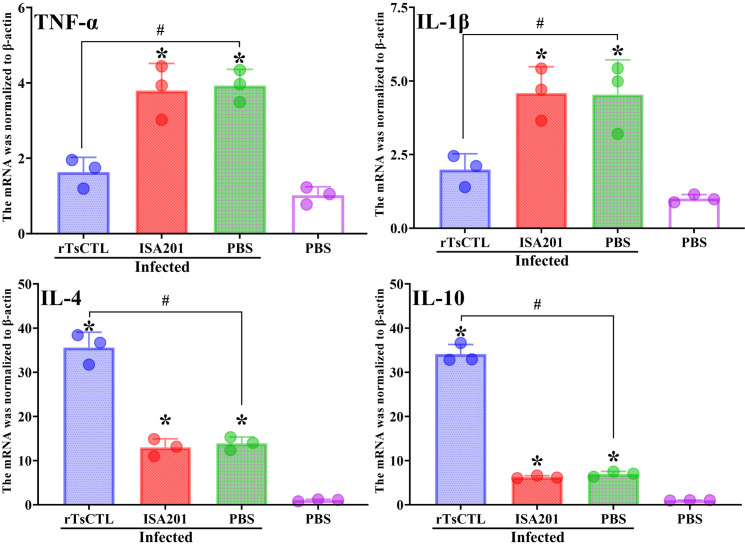
qPCR analysis of intestinal inflammatory cytokine mRNA transcription levels in immunized mice at 7 dpi. Total mRNA of intestinal tissue from immunized mice was extracted, and qPCR was performed to measure the transcription levels of TNF-α, IL-1β, IL-4 and IL-10 at 7 dpi. β-actin was used as an internal reference. Each test had three replicates. *P < 0.05 compared to the uninfected PBS groups; ^#^*P* < 0.05 contrast to the infected PBS group.

Total RNAs were obtained from intestinal tissue of infected mice at 7 dpi, the mRNA expression levels of intestinal inflammatory cytokines were assessed by qPCR. The results showed that after challenge, pro-inflammatory cytokines (TNF-α, IL-1β) transcription levels in rTsCTL immunized mice were overtly lower than the ISA201 and infected PBS groups (*F*_TNF-α_ = 17.60, *P* < 0.01; *F*_IL-1β_ = 8.080, *P* < 0.05) (Fig 12). But, anti-inflammatory cytokine (IL-4 and IL-10) transcription level in the rTsCTL group was significantly higher than the ISA201 and infected PBS groups (*F*_IL-4_ = 82.39, *F*_IL-10_ = 442.2, *P* < 0.0001). The results indicated that immunization with rTsCTL significantly reduced the pro-inflammatory factors (TNF-α, IL-1β) transcription and increased anti-inflammatory cytokines (IL-4, IL-10) transcription, consequently relieved enteric inflammatory reaction in rTsCTL-immunized mice after challenge.

### Muscle pathological change in infected mice

**Fig 13:**
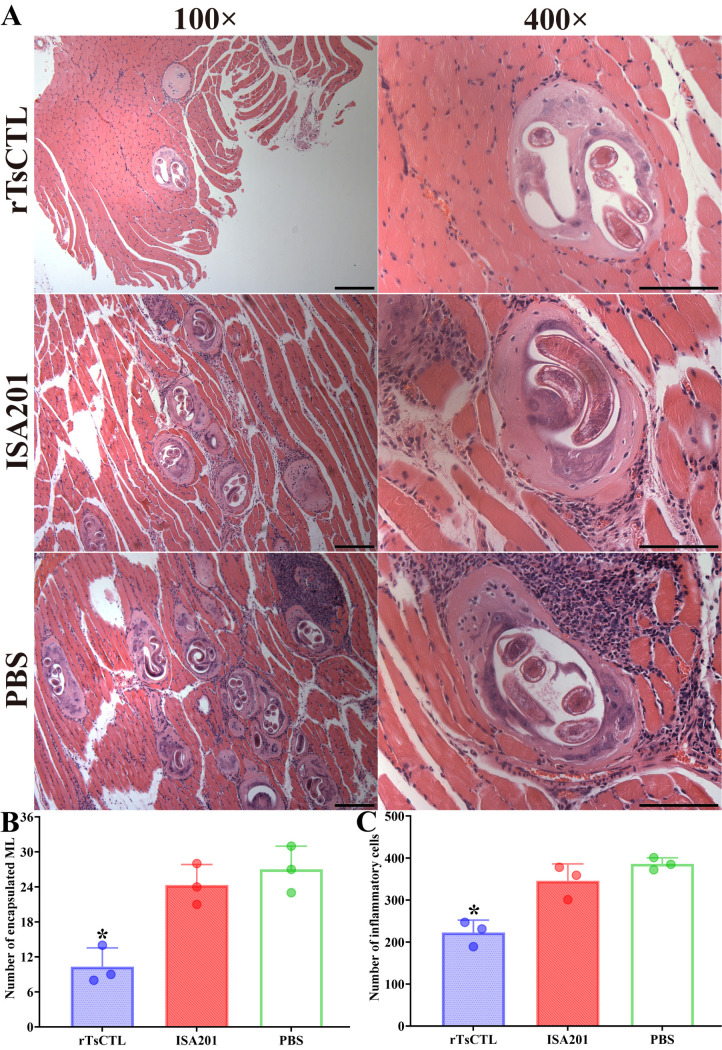
Pathological changes of mouse masseter muscle at 35 dpi. **A:** Muscle tissue section was dyed by HE and observed on microscopy. Mild inflammatory reaction and less encapsulated muscle larvae were observed in muscle section of rTsCTL immunized mice. Scale bars = 200 μm. **B:** Number of encapsulated muscle larvae in different groups of vaccinated mice. **C:** Number of inflammatory cells around encapsulated larvae in different groups of vaccinated mice. Each test had three replicates. *P < 0.05 compared to the ISA201 and PBS groups.

Pathological change of skeletal muscles from infected mice was observed at 35 dpi. In the rTsCTL group, muscle fibers were relative normal and uniform, and muscle cells were relative visible. However, the sarcolemma of the larval parasitized muscle fibers was severely destroyed in the muscle sections of the ISA201 and PBS groups, and more intense inflammation reaction was observed (Fig 13A). The number of encapsulated *T. spiralis* larvae of the rTsCTL group was distinctly lower than the ISA201 and PBS groups (*F* = 18.66, *P* = 0.0027) (Fig 13B). Additionally, the inflammatory infiltrative cells around the encapsulated larvae of the rTsCTL group were also significantly reduced compared to the ISA201 and PBS groups (*F* = 24.11, *P* = 0.0014) (Fig 13C).

## Discussion

C-type lectins (CTL) are a class of Ca^2+^-dependent lectins with a compact globular sugar recognition domain (carbohydrate recognition domain, CRD), and it is the most diverse and abundant family of lectins. CTL has the C-type lectin domain (CTLD) containing pattern recognition receptors. It binds to monosaccharides or oligosaccharides in a Ca^2+^-dependent manner to recognize the glycosyl molecules on the surface of cells [48]. CTL plays an important role in parasite adhesion, invasion and immune evasion. A CTL has been discovered in the excretory/secretory products from *Toxocara canis* infectious larvae. This lectin specifically attaches to receptors on the exterior of canine MDCK cells in a manner dependent on calcium in laboratory conditions [49]. The C-type lectin (CD209a) on host dendritic cells combined with glycoproteins on the surface of *Schistosoma* eggs, promoted schistosome juvenile invasion of the connective tissues of the host [50]. Therefore, the parasite CTL is likely to be a potential vaccine target molecule against parasite infection.

Previous study showed that a novel TsCTL was highly expressed at the IIL stage and located in the cuticle, stichosome and embryos of female adults; TsCTL was directly contacted and interacted with host intestinal epithelium at the early stage of *T. spiralis* infection [19]. TsCTL as a surface and secretory antigen was also early exposed to the host’s immune system, it could trigger the generation of specific anti-*Trichinella* IgG antibodies. Recent studies indicated that there was a specific binding between rTsCTL and syndecan-1 in Caco-2 cells and murine gut epithelium in vitro and in vivo, which activated the STAT3 pathway, reduced expression of TJs, damaged intestinal epithelium integrity, and mediated the IIL intrusion of intestinal mucosa [21,51]. Therefore, TsCTL might be a candidate molecule target of preventive vaccines against *T. spiralis* invasion and infection.

In this study, to further investigate whether rTsCTL induces immune protection, the mice were subcutaneously vaccinated by rTsCTL with the adjuvant ISA 201. The results revealed that the mice vaccinated with rTsCTL produced dramatically elevated specific anti-rTsCTL antibodies (serum IgG, IgG1/IgG2a and IgA, and gut sIgA). Immunization with rTsCTL also triggered systemic (spleen) and local gut mucosal (MLN and PP) cellular immune response, as demonstrated by an evident elevation of Th1 cytokine IFN-γ and Th2 cytokine IL-4. The mixed Th1/Th2 response acts a vital role in protective immunity against *T. spiralis* larva attack [27,31]. Intestinal sIgA plays a crucial part in gut mucosal immune response and prevents pathogen invasion from the gut mucosa. Most pathogens invade the host intestinal mucosal surfaces after they are ingested, and sIgA as the first natural barrier ensures the host get the protection. Intestinal sIgA against surface antigens of intestinal *T. spiralis* stages blocked parasites from invading gut epithelium through accelerating the enteral IIL and AW expulsion from the gut [13,47,52]. Passive transfer of anti-*Trichinella* IgA mediated *T. spiralis* excretion from murine gut after larval challenge [53]. Moreover, sIgA is involved in the Th2 immune response. IL-4 is the dominating cytokine in amplifying the IgA response, suggesting that elevated levels of IL-4 effectively enhanced the mucosal IgA response [6]. Additionally, intestinal sIgA also could inhibit the reproduction capacity (fecundity) of *T. spiralis* female adults [26,30]. Anti-*Trichinella* IgG is also contributed to the rapid ejectment of *T. spiralis* from the gut. Our results showed that the female fecundity (e.g., the NBL production/female in vitro for 72 h) of vaccinated mice was significantly inferior to the ISA 201 or PBS control mice, indicating that rTsCTL immunization generated an obvious protective immunity, impeded intestinal worm development and reduced the female adult fecundity.

A biased Th2-type immune response is a hallmark of helminth infection, and it has been demonstrated that intestinal worms had a regulatory mechanism that limits Th1 responses. IL-4 promotes the generation of IgE, mast cells, and mucus; it also prompts CD4 ^+^ T cells to mature into Th2 cells, increases the IFN-γ secretion, and suppresses the production of Th1 cytokines. Our ELISA results revealed that at 6 weeks after the first immunization, the secretion level of IFN-γ and IL-4 by spleen cells, MLN and PP in the rTsCTL group were remarkably increased, and further elevated following larval challenge, confirming that rTsCTL immunization elicited Th1/Th2-mixed cellular immune response. Th2-type immune reaction plays a primary role in combating intestinal nematode infection. The Th2-type immune responses mainly presented as an expansion of mast cells and goblet cells, elevation in mucus production, heightened levels of specific cytokines, histamine, and the generation of antibodies (IgG1 and IgE) [54]. Goblet cells, specialized in secreting mucus within the intestinal epithelium, play a crucial role in expelling worms from the gut by enhancing mucus secretion. The abundance of goblet cells and mucin secretion are directly related with the intensity of *T. spiralis* infection [27].

Mucins (including Muc2, Muc5ac and Muc5b), a glycoprotein secreted by goblet cells, are the primary constituent of mucus, forming a viscous and flexible gel-like layer [55]. Mucins play a vital role in the innate defense against intestinal nematode infections. The heightened secretion of Muc2 was found to be strongly linked to the worm expulsion from the intestines. Conversely, in mice lacking Muc2, the expulsion of *Trichuris muris* worms from the intestines was notably delayed. Muc5ac acts a crucial role in facilitating the intestinal nematode expulsion, with its presence being notably elevated in the cecum of mice who showed resistance to *Trichuris muris* infection. Conversely, in mice lacking Muc5ac, the ability to expel *T. muris* from the gastrointestinal tract was compromised, often resulting in persistent and chronic infections. The lack of Muc5ac led to a noticeable postponement in the ejection of two additional gastrointestinal nematodes (*T. spiralis* and *Nippostrongylus brasiliensis*) [56]. Our results showed that rTsCTL immunization significantly hampered larval invasion of gut mucosa, alleviated intestinal inflammation, and reduced the goblet cell number and mucin expression level in gut mucosa [13,32].

Intestinal epithelial cells act as a physical barrier and participate in intestinal mucosal immunity, resisting intestinal lumen antigens, toxins and harmful substances as the first line of defense [57]. Therefore, the balance between epithelial proliferation, injury, apoptosis, and inflammatory responses maintains the intestinal epithelial integrity to keep normal barrier function. As the predominant intercellular connections, tight junctions (TJs) play a prime role in regulating the permeability of intestinal epithelium and intestinal barrier function [58]. Occludin and claudins maintain cell polarity and intestinal epithelial barrier. Previous studies showed that overexpression or up-regulation of claudin-2 increased intestinal permeability and worsen colitis [59]. It has been showed that various cytokines (inflammatory factors, chemokines, tumor necrosis factors, and other signaling factors) affect the state of TJs and regulate enteral homeostasis and stress response in vivo and in vitro [60,61].The high expression of TNF-α increased the permeability of intestinal epithelium, and reduced the expression of occludin and ZO-1 [62]; whereas the pro-inflammatory cytokine IL-1β down-regulated the occludin expression and increased the permeability of Caco-2 cell monolayer [63].

In the present study, intestinal pathological results showed that at 1 week after challenge, less intestinal inflammation and relative normal intestinal villi were observed in rTsCTL-immunized mice. Moreover, intestinal villus width, goblet cell numbers and expression levels of Muc2 and Muc5ac were notably reduced in the rTsCTL-immunized mice. qPCR and Western blot results revealed that increased expression levels of TJs (occludin, claudin-1, E-cad), reduced claudin-2 expression and intestinal permeability were found in the rTsCTL immunized mice. Furthermore, in the rTsCTL-immunized mice, the levels of intestinal pro-inflammatory cytokines (TNF-α and IL-1β) were significantly reduced; whereas the expression of anti-inflammatory cytokines (IL-4 and IL-10) was notably increased. These findings demonstrated that rTsCTL immunization triggered an obvious mixed Th1/Th2 immune response both locally in the gut and systemically [5,25]. The protective immunity effectively hindered the larval invasion of intestine, alleviated gut inflammation, declined expression levels of mucins and pro-inflammatory cytokines; consequently improved intestinal epithelial integrity and enhanced intestinal mucosal barrier function [36,64].

After being orally challenged by the ML, compared with the ISA 201 or PBS alone group, the AW burdens at 7 dpi and ML burdens at 35 dpi of the rTsCTL-vaccinated mice were decreased by 51.69 and 48.19%, respectively. However, compared with the PBS group, mice vaccinated with ISA 201 adjuvant alone did not exhibit any significant reduction in intestinal AW and ML burdens. Vaccination of mice with rTsCTL induced an evident immune protection against larval infections by producing high levels of specific IgG and sIgA, IFN-γ and IL-4 cytokines [25]. The immune protection produced by rTsCTL vaccination may be involved in a combination of impeding larval invasion and development, expelling residual IIL and adult worms from the gut, decreasing female fecundity and ADCC-mediated killing on NBL and ML. Anti-*Trichinella* IgG antibodies effectively bind to the IIL outer cuticle, and formed a protective immune complex that acts as a physical barrier in larval anterior. This immune complex functions by physically obstructing direct contact between IIL and the gut epithelium, thereby preventing larval penetration into intestinal mucosa and partially impeding larval development [6,65,66]. To further assess the anti-*Trichinella* IgG mediated cytotoxicity, an in vitro ADCC test was carried out in this study. The results revealed that anti-*Trichinella* IgG antibodies facilitated and expedited the macrophages’ adherence and NBL damage, while the ADCC cytotoxicity was directly related to the concentration of anti-*Trichinella* antibodies. These results indicated that anti-*Trichinella* antibodies actively participated in the destruction and elimination of NBL through an ADCC mechanism [12]. Additionally, the protective role of IFN-γ against *T. spiralis* infection is mediated through macrophage’s activation and its enhancement of cytotoxic killing [67].

Additionally, the numbers of encapsulated larvae and inflammatory infiltrative cells surrounding the larvae in muscle sections were significantly reduced in rTsCTL immunized groups at 35 dpi, compared with the only ISA201 or PBS groups. The results demonstrated that rTsCTL vaccination effectively reduced larval burdens and alleviated inflammatory infiltration in the muscle tissues of infected mice. This phenomenon may be involved in the immunomodulatory effects of IL-10 produced by the vaccination, which suppressed inflammatory responses during the muscle stage of *T. spiralis* infection [68]. However, the infective ML was not fully eradicated from vaccinated animals. The protective immunity elicited by subcutaneous vaccination with rTsCTL was insufficient to effectively prevent and completely block *Trichinella* infection. The results showed that vaccination with a single *Trichinella* protein only led to partial immune protection against challenge infection. *T. spiralis* is a multicellular zoonotic parasitic nematode with a complex life cycle and each worm stage has its stage-specific antigens [39]. Therefore, to eliminate *Trichinella* larvae in food animals, a multivalent anti-*Trichinella* vaccine consisting of various protective epitopes needs to be developed in domestic food animals [69]. Previous studies have shown that oral administration of recombinant NC8-Tsgal vaccine and recombinant NC8/TsPPase DNA vaccines induced systemic mixed Th1/Th2 immune response and local intestinal mucosal response [5,27]; Other studies have shown that DNA vaccine could induce gut local IgA and systemic IgA response in mice, resulting in a protection against *Trichinella* infection [6,70]. Adjuvants have the ability to modulate cellular and humoral immune responses. In recent years, sugars and polysaccharides have been used as adjuvants of anti-*Trichinella* vaccines, and intestinal adult and muscle larva burden were significantly decreased in infected mice pre-treated with mannose [20]. Beta-glucan (BG) induced intestinal microbiota-dependent metabolites. The mucus layer was thickened and facilitated the intestinal *Trichinella* expulsion [71]. Another study indicated specific protective immunity against *Trichinella* challenge was enhanced with galactomannan as an adjuvant [32]. Therefore, the eradication of *T. spiralis* muscle larvae in food animals requires the development of additional vaccination strategies, such as further screening protective antigens, heterologous prime-boost vaccination, and novel adjuvants, and so on [13]. Furthermore, considering that pork serves as a primary source of human *Trichinella* infection, it is imperative from a veterinary perspective to implement the conclusive experiments on a domestic pig model in order to validate the eventual protective efficacy of anti-*Trichinella* vaccines.

In conclusion, vaccination of mice with rTsCTL produced a systemic Th1/Th2 mixed response and local enteral mucosal sIgA response. The vaccinated mice exhibited a significant immune protection, as demonstrated by female fecundity reduction, 51.69 and 48.19% reduction of enteral adult and muscle larva burdens following *T. spiralis* larval challenge. rTsCTL vaccination also alleviated gut inflammations, improved intestinal epithelial integrity and enhanced intestinal mucosal barrier function. The results indicated that TsCTL might be a novel candidate target molecule for anti-*Trichinella* vaccines.

## Supporting information

S1 Table The raw data for building the graphs in this study. (XLSX)

## References

[1] Rostami A, Gamble HR, Dupouy-Camet J, Khazan H, Bruschi F. Meat sources of infection for outbreaks of human trichinellosis. Food Microbiol. 2017;64:65–71. doi: 10.1016/j.fm.2016.12.012 28213036

[2] European Food Safety Authority (EFSA). European Centre for Disease Prevention and Control (ECDC) The European Union One Health 2022 Zoonoses report. EFSA J. 2023;21:e8442. doi: 10.2903/j.efsa.2023.8442 38089471 PMC10714251

[3] Zhang XZ, Wang ZQ, Cui J. Epidemiology of trichinellosis in the People’s Republic of China during 2009-2020. Acta Trop. 2022;229:106388. doi: 10.1016/j.actatropica.2022.106388 35231417

[4] Vasilev S, Mitic I, Mirilovic M, Plavsa D, Milakara E, Plavsic B, et al. *Trichinella* infection in Serbia from 2011 to 2020: a success story in the field of One Health. Epidemiol Infect. 2023;151:e20. doi: 10.1017/S0950268823000109 36655706 PMC9990384

[5] Hu CX, Xu YXY, Hao HN, Liu RD, Jiang P, Long SR, et al. Oral vaccination with recombinant *Lactobacillus plantarum* encoding *Trichinella spiralis* inorganic pyrophosphatase elicited a protective immunity in BALB/c mice. PLoS Negl Trop Dis. 2021;15(10):e0009865. doi: 10.1371/journal.pntd.0009865 34699522 PMC8547688

[6] Zhang XZ, Yue WW, Bai SJ, Hao HN, Song YY, Long SR, et al. Oral immunization with attenuated *Salmonella* encoding an elastase elicits protective immunity against *Trichinella spiralis* infection. Acta Trop. 2022;226:106263. doi: 10.1016/j.actatropica.2021.106263 34879232

[7] Hu CX, Zeng J, Yang DQ, Yue X, Dan Liu R, Long SR, et al. Binding of elastase-1 and enterocytes facilitates *Trichinella spiralis* larval intrusion of the host’s intestinal epithelium. Acta Trop. 2020;211:105592. doi: 10.1016/j.actatropica.2020.105592 32565198

[8] Song YY, Lu QQ, Han LL, Yan SW, Zhang XZ, Liu RD, et al. Proteases secreted by *Trichinella spiralis* intestinal infective larvae damage the junctions of the intestinal epithelial cell monolayer and mediate larval invasion. Vet Res. 2022;53(1):19. doi: 10.1186/s13567-022-01032-1 35255974 PMC8900307

[9] Ren HN, Zhuo TX, Bai SJ, Bai Y, Sun XY, Dan Liu R, et al. Proteomic analysis of hydrolytic proteases in excretory/secretory proteins from *Trichinella spiralis* intestinal infective larvae using zymography combined with shotgun LC-MS/MS approach. Acta Trop. 2021;216:105825. doi: 10.1016/j.actatropica.2021.105825 33421420

[10] Ortega-Pierres G, Vaquero-Vera A, Fonseca-Liñán R, Bermúdez-Cruz RM, Argüello-García R. Induction of protection in murine experimental models against *Trichinella spiralis*: an up-to-date review. J Helminthol. 2015;89(5):526–39. doi: 10.1017/S0022149X15000140 25761655

[11] Sun GG, Ren HN, Liu RD, Song YY, Qi X, Hu CX, et al. Molecular characterization of a putative serine protease from *Trichinella spiralis* and its elicited immune protection. Vet Res. 2018;49(1):59. doi: 10.1186/s13567-018-0555-5 30001738 PMC6043985

[12] Yue X, Sun XY, Liu F, Hu CX, Bai Y, Da Yang Q, et al. Molecular characterization of a *Trichinella spiralis* serine proteinase. Vet Res. 2020;51(1):125. doi: 10.1186/s13567-020-00847-0 32988413 PMC7520982

[13] Bai SJ, Han LL, Liu RD, Long SR, Zhang X, Cui J, et al. Oral vaccination of mice with attenuated *Salmonella* encoding *Trichinella spiralis* calreticulin and serine protease 1.1 confers protective immunity in BALB/c mice. PLoS Negl Trop Dis. 2022;16(11):e0010929. doi: 10.1371/journal.pntd.0010929 36445875 PMC9707759

[14] Dambuza IM, Brown GD. C-type lectins in immunity: recent developments. Curr Opin Immunol. 2015;32:21–7. doi: 10.1016/j.coi.2014.12.002 25553393 PMC4589735

[15] Li K, Underhill DM. C-type lectin receptors in phagocytosis. Curr Top Microbiol Immunol. 2020;429:1–18. doi: 10.1007/82_2020_198 32060644

[16] Shi W, Xue C, Su X-Z, Lu F. The roles of galectins in parasitic infections. Acta Trop. 2018;177:97–104. doi: 10.1016/j.actatropica.2017.09.027 28986248 PMC5672833

[17] Njiri OA, Zhang X, Zhang Y, Wu B, Jiang L, Li Q, et al. CD209 C-type lectins promote host invasion, dissemination, and infection of *Toxoplasma gondii*. Front Immunol. 2020;11:656. doi: 10.3389/fimmu.2020.00656 32391004 PMC7190871

[18] Ludington JG, Ward HD. The *Cryptosporidium parvum* C-type lectin CpClec mediates infection of intestinal epithelial cells via interactions with sulfated proteoglycans. Infect Immun. 2016;84(5):1593–602. doi: 10.1128/IAI.01410-15 26975991 PMC4862729

[19] Hao HN, Song YY, Ma KN, Wang BN, Long SR, Liu RD, et al. A novel C-type lectin from *Trichinella spiralis* mediates larval invasion of host intestinal epithelial cells. Vet Res. 2022;53(1):85. doi: 10.1186/s13567-022-01104-2 36258242 PMC9580147

[20] Hao HN, Lu QQ, Wang Z, Li YL, Long SR, Dan Liu R, et al. Mannose facilitates *Trichinella spiralis* expulsion from the gut and alleviates inflammation of intestines and muscles in mice. Acta Trop. 2023;241:106897. doi: 10.1016/j.actatropica.2023.106897 36931335

[21] Wang Z, Lu QQ, Weng MM, Li YL, Han LL, Song YY, et al. Binding of *Trichinella spiralis* C-type lectin with syndecan-1 on intestinal epithelial cells mediates larval invasion of intestinal epithelium. Vet Res. 2023;54(1):86. doi: 10.1186/s13567-023-01217-2 37784173 PMC10546719

[22] Sun GG, Song YY, Jiang P, Ren HN, Yan SW, Han Y, et al. Characterization of a *Trichinella spiralis* putative serine protease. Study of its potential as sero-diagnostic tool. PLoS Negl Trop Dis. 2018;12(5):e0006485. doi: 10.1371/journal.pntd.0006485 29758030 PMC5967804

[23] Xu J, Yang F, Yang DQ, Jiang P, Liu RD, Zhang X, et al. Molecular characterization of *Trichinella spiralis* galectin and its participation in larval invasion of host’s intestinal epithelial cells. Vet Res. 2018;49(1):79. doi: 10.1186/s13567-018-0573-3 30068382 PMC6071371

[24] Guo KX, Bai Y, Ren HN, Sun XY, Song YY, Liu RD, et al. Characterization of a *Trichinella spiralis* aminopeptidase and its participation in invasion, development and fecundity. Vet Res. 2020;51(1):78. doi: 10.1186/s13567-020-00805-w 32539772 PMC7296678

[25] Zeng J, Zhang XZ, Zhang R, Yan SW, Song YY, Long SR, et al. Vaccination of mice with recombinant novel aminopeptidase P and cathepsin X alone or in combination induces protective immunity against *Trichinella spiralis* infection. Acta Trop. 2021;224:106125. doi: 10.1016/j.actatropica.2021.106125 34508714

[26] Zhang XZ, Sun XY, Bai Y, Song YY, Hu CX, Li X, et al. Protective immunity in mice vaccinated with a novel elastase-1 significantly decreases *Trichinella spiralis* fecundity and infection. Vet Res. 2020;51(1):43. doi: 10.1186/s13567-020-00767-z 32169101 PMC7071723

[27] Xu YXY, Zhang XZ, Weng MM, Cheng YK, Liu RD, Long SR, et al. Oral immunization of mice with recombinant *Lactobacillus plantarum* expressing a *Trichinella spiralis* galectin induces an immune protection against larval challenge. Parasit Vectors. 2022;15(1):475. doi: 10.1186/s13071-022-05597-w 36539832 PMC9764493

[28] Bermúdez-Cruz RM, Fonseca-Liñán R, Grijalva-Contreras LE, Mendoza-Hernández G, Ortega-Pierres MG. Proteomic analysis and immunodetection of antigens from early developmental stages of *Trichinella spiralis*. Vet Parasitol. 2016;231:22–31. doi: 10.1016/j.vetpar.2016.06.029 27396501

[29] Qi X, Han Y, Jiang P, Yue X, Ren HN, Sun GG, et al. Oral vaccination with *Trichinella spiralis* DNase II DNA vaccine delivered by attenuated *Salmonella* induces a protective immunity in BALB/c mice. Vet Res. 2018;49(1):119. doi: 10.1186/s13567-018-0614-y 30518422 PMC6280372

[30] Sun GG, Lei JJ, Ren HN, Zhang Y, Guo KX, Long SR, et al. Intranasal immunization with recombinant *Trichinella spiralis* serine protease elicits protective immunity in BALB/c mice. Exp Parasitol. 2019;201:1–10. doi: 10.1016/j.exppara.2019.04.006 31004570

[31] Pompa-Mera EN, Arroyo-Matus P, Ocaña-Mondragón A, González-Bonilla CR, Yépez-Mulia L. Protective immunity against enteral stages of *Trichinella spiralis* elicited in mice by live attenuated *Salmonella* vaccine that secretes a 30-mer parasite epitope fused to the molecular adjuvant C3d-P28. Res Vet Sci. 2014;97(3):533–45. doi: 10.1016/j.rvsc.2014.09.010 25311159

[32] Zhang R, Zhang XZ, Guo X, Han LL, Wang BN, Zhang X, et al. The protective immunity induced by *Trichinella spiralis* galectin against larval challenge and the potential of galactomannan as a novel adjuvant. Res Vet Sci. 2023;165:105075. doi: 10.1016/j.rvsc.2023.105075 37931574

[33] Gajadhar AA, Noeckler K, Boireau P, Rossi P, Scandrett B, Gamble HR. International Commission on trichinellosis: recommendations for quality assurance in digestion testing programs for *Trichinella*. Food Waterborne Parasitol. 2019;16:e00059. doi: 10.1016/j.fawpar.2019.e00059 32095629 PMC7033998

[34] Hu YY, Zhang R, Yan SW, Yue WW, Zhang JH, Liu RD, et al. Characterization of a novel cysteine protease in *Trichinella spiralis* and its role in larval intrusion, development and fecundity. Vet Res. 2021;52(1):113. doi: 10.1186/s13567-021-00983-1 34446106 PMC8390047

[35] Marincola Smith P, Choksi YA, Markham NO, Hanna DN, Zi J, Weaver CJ, et al. Colon epithelial cell TGFβ signaling modulates the expression of tight junction proteins and barrier function in mice. Am J Physiol Gastrointest Liver Physiol. 2021;320(6):G936–57. doi: 10.1152/ajpgi.00053.2021 33759564 PMC8285585

[36] Song YY, Zhang XZ, Wang BN, Cheng YK, Guo X, Zhang X, et al. A novel *Trichinella spiralis* serine proteinase disrupted gut epithelial barrier and mediated larval invasion through binding to RACK1 and activating MAPK/ERK1/2 pathway. PLoS Negl Trop Dis. 2024;18(1):e0011872. doi: 10.1371/journal.pntd.0011872 38190388 PMC10798628

[37] Wang R, Zhang Y, Zhen J, Zhang J, Pang Z, Song X, et al. Effects of exosomes derived from *Trichinella spiralis* infective larvae on intestinal epithelial barrier function. Vet Res. 2022;53(1):87. doi: 10.1186/s13567-022-01108-y 36273217 PMC9587624

[38] Han LL, Lu QQ, Zheng WW, Li YL, Song YY, Zhang XZ, et al. A novel trypsin of *Trichinella spiralis* mediates larval invasion of gut epithelium via binding to PAR2 and activating ERK1/2 pathway. PLoS Negl Trop Dis. 2024;18(1):e0011874. doi: 10.1371/journal.pntd.0011874 38166153 PMC10786404

[39] Ren HN, Liu RD, Song YY, Zhuo TX, Guo KX, Zhang Y, et al. Label-free quantitative proteomic analysis of molting-related proteins of *Trichinella spiralis* intestinal infective larvae. Vet Res. 2019;50(1):70. doi: 10.1186/s13567-019-0689-0 31547875 PMC6757440

[40] Hu CX, Zeng J, Hao HN, Xu YXY, Liu F, Liu RD, et al. Biological properties and roles of a *Trichinella spiralis* inorganic pyrophosphatase in molting and developmental process of intestinal larval stages. Vet Res. 2021;52(1):6. doi: 10.1186/s13567-020-00877-8 33413587 PMC7791673

[41] Liu RD, Meng XY, Li CL, Long SR, Cui J, Wang ZQ. Molecular characterization and determination of the biochemical properties of cathepsin L of *Trichinella spiralis*. Vet Res. 2022;53(1):48. doi: 10.1186/s13567-022-01065-6 35739604 PMC9229914

[42] Bardi GT, Smith MA, Hood JL. Melanoma exosomes promote mixed M1 and M2 macrophage polarization. Cytokine. 2018;105:63–72. doi: 10.1016/j.cyto.2018.02.002 29459345 PMC5857255

[43] Xu J, Liu RD, Bai SJ, Hao HN, Yue WW, Xu YXY, et al. Molecular characterization of a *Trichinella spiralis* aspartic protease and its facilitation role in larval invasion of host intestinal epithelial cells. PLoS Negl Trop Dis. 2020; 14:e0008269. doi: 10.1371/journal.pntd.0008269 32339171 PMC7205320

[44] Song YY, Zhang XZ, Wang BN, Weng MM, Zhang ZY, Guo X, et al. Molecular characterization of a novel serine proteinase from *Trichinella spiralis* and its participation in larval invasion of gut epithelium. PLoS Negl Trop Dis. 2023;17(9):e0011629. doi: 10.1371/journal.pntd.0011629 37695792 PMC10513378

[45] Hu CX, Jiang P, Yue X, Zeng J, Zhang XZ, Song YY, et al. Molecular characterization of a *Trichinella spiralis* elastase-1 and its potential as a diagnostic antigen for trichinellosis. Parasit Vectors. 2020;13(1):97. doi: 10.1186/s13071-020-3981-y 32093735 PMC7041205

[46] Han LL, Lu QQ, Li YL, Zheng WW, Ren P, Liu RD, et al. Application of a recombinant novel trypsin from *Trichinella spiralis* for serodiagnosis of trichinellosis. Parasit Vectors. 2024;17(1):9. doi: 10.1186/s13071-023-06067-7 38178167 PMC10768479

[47] Lei JJ, Hu YY, Liu F, Yan SW, Liu RD, Long SR, et al. Molecular cloning and characterization of a novel peptidase from *Trichinella spiralis* and protective immunity elicited by the peptidase in BALB/c mice. Vet Res. 2020;51(1):111. doi: 10.1186/s13567-020-00838-1 32891183 PMC7487599

[48] Zelensky AN, Gready JE. The C-type lectin-like domain superfamily. FEBS J. 2005;272(24):6179–217. doi: 10.1111/j.1742-4658.2005.05031.x 16336259

[49] Loukas A, Doedens A, Hintz M, Maizels RM. Identification of a new C-type lectin, TES-70, secreted by infective larvae of *Toxocara canis*, which binds to host ligands. Parasitology. 2000;121 Pt 5:545–54. doi: 10.1017/s0031182099006721 11128806

[50] Kalantari P, Bunnell SC, Stadecker MJ. The C-type lectin receptor-driven, Th17 cell-mediated severe pathology in schistosomiasis: not all immune responses to helminth parasites are Th2 dominated. Front Immunol. 2019;10:26. doi: 10.3389/fimmu.2019.00026 30761125 PMC6363701

[51] Wang BN, Zhang XZ, Cong PK, Zheng WW, Wu JY, Long SR, et al. *Trichinella spiralis* C-type lectin mediates larva invasion of gut mucosa via binding to syndecan-1 and damaging epithelial integrity in mice. Int J Biol Macromol. 2024;280(Pt 4):135958. doi: 10.1016/j.ijbiomac.2024.135958 39322156

[52] Li JF, Guo KX, Qi X, Lei JJ, Han Y, Yan SW, et al. Protective immunity against *Trichinella spiralis* in mice elicited by oral vaccination with attenuated *Salmonella*-delivered TsSP1.2 DNA. Vet Res. 2018;49(1):87. doi: 10.1186/s13567-018-0582-2 30189894 PMC6127904

[53] Inaba T, Sato H, Kamiya H. Monoclonal IgA antibody-mediated expulsion of *Trichinella* from the intestine of mice. Parasitology. 2003;126(Pt 6):591–8. doi: 10.1017/s003118200300310x 12866798

[54] Saracino MP, Vila CC, Cohen M, Gentilini MV, Falduto GH, Calcagno MA, et al. Cellular and molecular changes and immune response in the intestinal mucosa during *Trichinella spiralis* early infection in rats. Parasit Vectors. 2020;13(1):505. doi: 10.1186/s13071-020-04377-8 33023672 PMC7539519

[55] Coakley G, Harris NL. The intestinal epithelium at the forefront of host-helminth interactions. Trends Parasitol. 2020;36(9):761–72. doi: 10.1016/j.pt.2020.07.002 32713764

[56] Hasnain SZ, Evans CM, Roy M, Gallagher AL, Kindrachuk KN, Barron L, et al. Muc5ac: a critical component mediating the rejection of enteric nematodes. J Exp Med. 2011;208(5):893–900. doi: 10.1084/jem.20102057 21502330 PMC3092342

[57] Pitman RS, Blumberg RS. First line of defense: the role of the intestinal epithelium as an active component of the mucosal immune system. J Gastroenterol. 2000;35(11):805–14. doi: 10.1007/s005350070017 11085489

[58] Zihni C, Mills C, Matter K, Balda MS. Tight junctions: from simple barriers to multifunctional molecular gates. Nat Rev Mol Cell Biol. 2016;17(9):564–80. doi: 10.1038/nrm.2016.80 27353478

[59] Raju P, Shashikanth N, Tsai P-Y, Pongkorpsakol P, Chanez-Paredes S, Steinhagen PR, et al. Inactivation of paracellular cation-selective claudin-2 channels attenuates immune-mediated experimental colitis in mice. J Clin Invest. 2020;130(10):5197–208. doi: 10.1172/JCI138697 32516134 PMC7524482

[60] Walsh SV, Hopkins AM, Nusrat A. Modulation of tight junction structure and function by cytokines. Adv Drug Deliv Rev. 2000;41(3):303–13. doi: 10.1016/s0169-409x(00)00048-x 10854688

[61] Nunes T, Bernardazzi C, de Souza HS. Cell death and inflammatory bowel diseases: apoptosis, necrosis, and autophagy in the intestinal epithelium. Biomed Res Int. 2014;2014:218493. doi: 10.1155/2014/218493 25126549 PMC4121991

[62] Haque M, Kaminsky L, Abdulqadir R, Engers J, Kovtunov E, Rawat M, et al. *Lactobacillus acidophilus* inhibits the TNF-α-induced increase in intestinal epithelial tight junction permeability via a TLR-2 and PI3K-dependent inhibition of NF-κB activation. Front Immunol. 2024;15:1348010. doi: 10.3389/fimmu.2024.1348010 39081324 PMC11286488

[63] Al-Sadi RM, Ma TY. IL-1beta causes an increase in intestinal epithelial tight junction permeability. J Immunol. 2007;178(7):4641–9. doi: 10.4049/jimmunol.178.7.4641 17372023 PMC3724221

[64] Ma KN, Zhang Y, Zhang ZY, Wang BN, Song YY, Han LL, et al. *Trichinella spiralis* galectin binding to toll-like receptor 4 induces intestinal inflammation and mediates larval invasion of gut mucosa. Vet Res. 2023;54(1):113. doi: 10.1186/s13567-023-01246-x 38012694 PMC10680189

[65] McVay CS, Bracken P, Gagliardo LF, Appleton J. Antibodies to tyvelose exhibit multiple modes of interference with the epithelial niche of *Trichinella spiralis*. Infect Immun. 2000;68(4):1912–8. doi: 10.1128/IAI.68.4.1912-1918.2000 10722582 PMC97366

[66] Yan SW, Cheng YK, Lu QQ, Zhang R, Dan Liu R, Long SR, et al. Characterization of a novel dipeptidyl peptidase 1 of *Trichinella spiralis* and its participation in larval invasion. Acta Trop. 2024;249:107076. doi: 10.1016/j.actatropica.2023.107076 37977254

[67] Patel N, Kreider T, Urban JF Jr, Gause WC. Characterisation of effector mechanisms at the host: parasite interface during the immune response to tissue-dwelling intestinal nematode parasites. Int J Parasitol. 2009;39(1):13–21. doi: 10.1016/j.ijpara.2008.08.003 18804113 PMC2842902

[68] Fabre MV, Beiting DP, Bliss SK, Appleton JA. Immunity to *Trichinella spiralis* muscle infection. Vet Parasitol. 2009;159(3–4):245–8. doi: 10.1016/j.vetpar.2008.10.051 19070961 PMC3449155

[69] Wang L, Wang X, Bi K, Sun X, Yang J, Gu Y, et al. Vaccination with a paramyosin-based multi-epitope vaccine elicits significant protective immunity against *Trichinella spiralis* infection in mice. Front Microbiol. 2017;8:1475. doi: 10.3389/fmicb.2017.01475 28824599 PMC5540943

[70] Wang L, Wang X, Bi K, Sun X, Yang J, Gu Y, et al. Oral vaccination with attenuated *Salmonella typhimurium*-delivered TsPmy DNA vaccine elicits protective immunity against *Trichinella spiralis* in BALB/c mice. PLoS Negl Trop Dis. 2016;10(9):e0004952. doi: 10.1371/journal.pntd.0004952 27589591 PMC5010209

[71] Jin X, Liu Y, Wang J, Wang X, Tang B, Liu M, et al. β-Glucan-triggered *Akkermansia muciniphila* expansion facilitates the expulsion of intestinal helminth via TLR2 in mice. Carbohydr Polym. 2022;275:118719. doi: 10.1016/j.carbpol.2021.118719 34742442

